# Effects of Wearing an N95 Respirator or Cloth Mask Among Adults at Peak Exercise: A Randomized Crossover Trial

**DOI:** 10.1001/jamanetworkopen.2021.15219

**Published:** 2021-06-30

**Authors:** Matthew Kampert, Tamanna Singh, Debasis Sahoo, Xiaozhen Han, Erik H. Van Iterson

**Affiliations:** 1 Center of Sports Medicine, Department of Orthopaedics, Orthopaedic and Rheumatology Institute, Cleveland Clinic, Cleveland, Ohio; 2 Center of Obesity and Medical Weight Loss, Endocrinology and Metabolism Institute Cleveland Clinic, Cleveland, Ohio; 3 Section of Clinical Cardiology, Miller Family Heart, Vascular and Thoracic Institute, Robert and Suzanne Tomsich Department of Cardiovascular Medicine, Cleveland Clinic, Cleveland, Ohio; 4 Department of Pulmonary and Critical Care Medicine, Respiratory Institute, Cleveland Clinic, Cleveland, Ohio; 5 Department of Quantitative Health Sciences, Cleveland Clinic, Cleveland, Ohio; 6 Section of Preventive Cardiology and Rehabilitation, Robert and Suzanne Tomsich Department of Cardiovascular Medicine, Miller Family Heart, Vascular and Thoracic Institute, Cleveland Clinic, Cleveland, Ohio

## Abstract

This randomized crossover trial examines the effects of wearing a cloth mask or N95 respirator vs no mask at peak exercise among healthy, active adults.

## Introduction

For the majority of apparently healthy adults, mask wearing while at rest or during activities of daily living is recommended to be safe and effective for reducing the risk of person-to-person airborne transmission of SARS-Cov-2.^1,2^ Original research has yet to find evidence showing an effect of mask wearing on clinical indicators of exercise safety.^3^ In this study, we tested whether mask wearing during exercise stress testing (EST) to peak exhaustion provokes clinically indicated safety concerns.

## Methods

In this randomized crossover trial (ClinicalTrials.gov Identifier: NCT04415879), 20 never-smoker, apparently healthy, recreationally active men and women participated in treadmill EST under each of the experimental conditions: no mask, N95 (3M 8200 N95 respirator), and cloth mask (Boco Gear PM2.5 activated carbon filter). A random number generator determined the order whereby experimental conditions would be performed. Participants provided voluntary verbal and written informed consent during study enrollment and prior to EST.

The Cleveland Clinic institutional review board reviewed and approved this study in accordance with the Declaration of Helsinki. The trial protocol is available in Supplement 1. The design and execution of this study also followed the Consolidated Standards of Reporting Trials (CONSORT) reporting guideline for crossover trials. A full description of the study methods, including the CONSORT flow diagram, can be viewed in the eAppendix of Supplement 2.

Individualized EST had participants self-select a constant treadmill belt speed that would be used across experimental conditions. Treadmill grade always increased from 0.0% to 2.0% at minute 2; and thereafter by 1.0% each minute until achieving peak exhaustion. Heart rate, rating of perceived exertion, and oxyhemoglobin saturation measurements were acquired throughout EST. Peak exercise oxygen uptake (V̇O_2_) was derived based on the Fitness Registry and the Importance of Exercise National Database equation using treadmill belt speed and grade. The subjective experience of wearing or not wearing a mask was also evaluated immediately postexercise using a perceptions instrument questionnaire. Established clinical indicators were used to evaluate exercise safety.^3^

The independent experimental condition effect on continuous variables was tested using mixed-effects analysis of variance modeling. The randomized EST order was set as a random effect in models. Two-tailed significance was determined using an alpha level set at .05. Statistical analysis was performed using SAS statistical software version 9.4 (SAS Institute) from October to December 2020.

## Results

Of 20 participants, there were 9 women (45%); the mean (SD) age and mean (SD) body mass index (calculated as weight in kilograms divided by height in meters squared) for women in the sample were 35 (11) years and 25.1 (4.2), respectively, which did not differ significantly from the men in the sample (39 [11] years and 25.0 [2.4], respectively). Performing EST with a mask yielded lower peak V̇O_2_ and heart rates as compared with no mask. Regardless of experimental condition, no participant demonstrated a clinical indication requiring EST termination prior to voluntary cessation associated with the achievement of peak exhaustion.

Univariate comparisons of physiological responses to EST in the Table consistently demonstrated exercise tolerance was the highest during the EST and no mask trial. However, symptoms were consistently severest during EST with a mask (eg, mean [SD] exercise duration was 591 [145] seconds without a mask vs 548 [147] seconds with a cloth mask vs 545 [141] seconds with an N95 mask; analysis of variation [ANOVA] *P* = .047). Perceived breathing resistance was the strongest symptom and unique to EST with a mask (eg, the median [interquartile range {IQR}] scores for subjective responses [0-10, with higher scores indicating worse symptoms] for breathing resistance were 0.0 [0.0-0.0] without a mask vs 7.0 [5.3-8.0] with a cloth mask vs 7.0 [5.5-8.3] with an N95 mask; ANOVA *P* < .001; the median [IQR] scores for feeling humid were 0.0 [0.0-1.0] without a mask vs 6.3 [3.5-7.8] with a cloth mask vs 7.0 [5.5-8.0] with an N95 mask; ANOVA *P* < .001). Adjusting comparisons of peak V̇O_2_ for perceived breathing resistance resulted in no significant experimental condition main effect (Figure).

**Table.** Exercise Physiological and Subjective Responses

Variable	Mean (SD)			*P* value^a^			
	No mask	Cloth mask	N95	ANOVA	No mask vs cloth mask	No mask vs N95	N95 vs cloth mask
Exercise duration, s	591 (145)	548 (147)	545 (141)	.047	.11	.10	.96
Peak V̇O_2_, mL/kg/min^b^	39.0 (8.9)	38.2 (8.7)	38.1 (8.6)	.009	.04	.01	.83
Peak V̇O_2_, % of estimated	103 (19)	101 (18)	101 (19)	.006	.02	.01	.91
Corrected peak V̇O_2_, mL/kg/min^c^	39.0 (8.9)	37.6 (8.8)	37.2 (8.4)	.03	.13	.04	.91
Peak METS, % of estimated	100 (20)	98 (20)	98 (20)	.006	.03	.01	.88
Peak Q, L/min^d^	19.3 (4.7)	19.4 (4.7)	19.3 (4.8)	.93	NA	NA	NA
Peak Ca-vO_2_, mL/dL^d^	15.1 (1.2)	14.8 (1.3)	14.8 (1.1)	.02	.03	.047	.98
Peak absolute difference in SpO_2_, %^e^	2.1 (3.1)	5.6 (3.6)	5.1 (4.0)	.005	.01	.01	>.99
Peak HR, bpm	177 (10)	174 (10)	174 (9)	.006	.02	.009	.92
Peak HR, % estimated	97 (6)	95 (7)	95 (5)	.004	.02	.005	.85
Peak RPE^f^ 0-10	9.6 (0.9)	9.3 (1.0)	9.5 (0.8)	.09	NA	NA	NA
HR*k*, bpm/m^g^	5.6 (1.4)	5.8 (2.0)	5.9 (1.6)	.43	NA	NA	NA
Submax HR, bpm^h^	144 (13)	143 (11)	144 (13)	.89	NA	NA	NA
Submax absolute difference in SpO_2_, %^e^	0.2 (1.2)	1.3 (2.0)	1.2 (1.2)	.04	.07	.09	>.99
Subjective responses at peak exercise, median (IQR)^i^							
Humid	0.0 (0.0-1.0)	6.3 (3.5-7.8)	7.0 (5.5-8.0)	<.001	<.001	<.001	.96
Hot	0.0 (0.0-1.5)	6.3 (3.3-7.5)	7.0 (5.0-8.0)	<.001	<.001	<.001	.53
Breathe resist	0.0 (0.0-0.0)	7.0 (5.3-8.0)	7.0 (5.5-8.3)	<.001	<.001	<.001	.99
Itchy	0.0 (0.0-0.0)	0.0 (0.0-0.5)	0.5 (0.0-1.0)	<.001	.04	<.001	.18
Tight	0.0 (0.0-0.0)	0.0 (0.0-2.0)	2.0 (0.5-3.5)	<.001	.003	<.001	.04
Salty	0.0 (0.0-0.0)	0.0 (0.0-0.5)	0.0 (0.0-1.0)	<.001	.051	<.001	.21
Unfit	0.0 (0.0-0.0)	1.0 (0.0-3.0)	1.0 (0.0-1.0)	<.001	<.001	.004	.52
Odor	0.0 (0.0-0.0)	0.3 (0.0-0.5)	1.0 (0.0-1.5)	<.001	.001	<.001	.06
Fatigue	4.8 (2.0-7.0)	5.8 (3.0-7.8)	7.5 (5.0-9.0)	.19	NA	NA	NA
Overall discomfort	3.0 (1.5-5.0)	4.8 (4.0-6.8)	5.0 (3.0-6.3)	.002	.001	.10	.15

Abbreviations: SpO_2_, oxygen saturation; ANOVA, analysis of variance; bpm, beats per minute; Ca-vO_2_, arterio-venous oxygen content difference; HR, heart rate; HR*k*, heart rate–rate of reaction constant; IQR, interquartile range; METS, metabolic equivalents of task performed; NA, not applicable for pairwise testing due to no significant overall effect of mask condition; RPE, rating of perceived exertion; Q, exercise cardiac output; V̇O_2_, exercise oxygen uptake.

^a^ Unadjusted mixed effect ANOVA *P* values in the table represent the overall effect of mask condition. In the table, pairwise testing and *P* values reflect post hoc corrections for multiple comparison testing.

^b^ Peak V̇O_2_ was estimated using terminal treadmill belt speed and grade as speed × (0.17 + grade × 0.79) + 3.5.

^c^ A peak V̇O_2_ corrected (C. peak V̇O_2_) for the change in observed oxygen saturation that occurred in cloth mask and N95 conditions as compared to no mask is also reported (see Supplement 2 for details).

^d^ Both Q and Ca-vO_2_ were estimated and corrected for observed oxygen saturation (see Supplement 2 for details).

^e^ Absolute difference in SpO_2_ is the absolute difference in oxygen saturation via pulse oximetry between rest and either stage 2 of exercise or peak exercise.

^f^ RPE is on a scale from 0 to 10 with higher scores indicating higher perceived exertion.

^g^ HR*k* is based on zeroth order model (see Supplement 2 for details).

^h^ Submax HR reflects the observed HR corresponding to the stage 2 of exercise which immediately followed the initial 0.0% grade stage.

^i^ Unit of measurement for subjective variables is on a 0 to 10 scale, where 0 is no severity and 10 is maximal severity.

**Figure.**
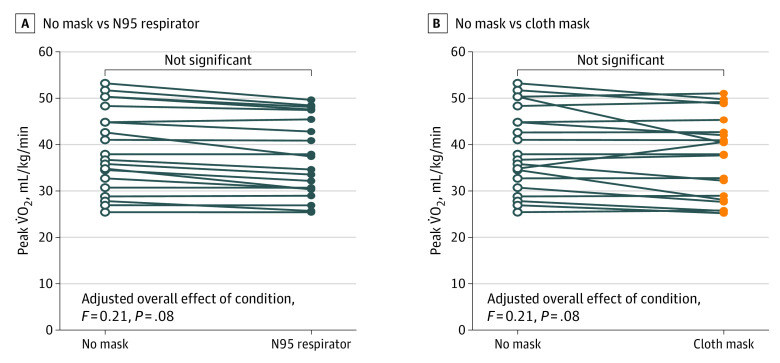
The Mixed-Effects Model Overall Main Effect of Mask Condition on Peak Exercise Oxygen Uptake (Peak V̇O_2_) Adjusted for the Covariate Effect of Perception of Breathing Resistance Reported peak V̇O_2_ for mask conditions reflect rest-to-peak exercise changes in oxygen saturation that occurred as compared with the no mask condition.

## Discussion

This crossover trial found that perceived breathing resistance at peak exercise is uniquely and significantly elevated when EST is performed while wearing a mask. Performing EST with a mask yielded lower peak V̇O_2_ and heart rates as compared with no mask. However, each experimental condition resulted in peak exercise values that generally remained within normal limits, and no EST required termination due to clinically indicated safety concerns. Thus, although it is possible that wearing a mask exerted a physical limitation on exercise capacity, the clinical relevance of such a possibility is not supported by these data.

A limitation of this study was the lack of exercise ventilation and gas exchange data collected during EST. This limited our ability to potentially identify where a major physiological limitation to exercise occurred during masked trials. However, having access to these data would likely not have changed the conclusion that performing EST with a mask is generally clinically safe but likely to provoke exaggerated symptoms. The subjective experience of high breathing resistance should not be overlooked as being potentially impactful to reducing how well individuals are able to perceptually tolerate vigorous-to-peak intensity exercise.

## Notes

Supplement 1. Trial Protocol

Supplement 2. **eAppendix.** Supplemental Methods

Supplement 3. Data Sharing Statement
