# Supplementary material for: Effects of Wearing an N95 Respirator or Cloth Mask Among Adults at Peak Exercise: A Randomized Crossover Trial
Source: JAMA Netw Open. 2021 Jun 30;4(6):e2115219. doi: 10.1001/jamanetworkopen.2021.15219 (PMC8246308; doi:10.1001/jamanetworkopen.2021.15219)
Supplement: Supplement 1. — Trial Protocol [file jamanetwopen-e2115219-s001.pdf]

Effects of a N95 Respirator vs Cloth Mask on Exercise Capacity During Treadmill Running.

Fellow: Matthew Kampert, MS, DO, RCEP

Mentor: Debasis Sahoo, MD

Department: Pulmonary Institute

TABLE OF CONTENTS

1. TITLE PAGE 1

2. BACKGROUND 3

3. SIGNIFICANCE 5

4 HYPOTHESIS 7

5. SPECIFIC AIMS 7

6. EXPERIMENTAL DESIGN & METHODS 7

7. STATISTICAL ANALYSIS 10

8. PROJECT TIMELINE 10

9. PERSONNEL 10

10. REFRENCES 10

11. APPENDIX 11

Background

The Centers for Disease Control and Prevention (CDC) state that the coronavirus disease 2019 (COVID-19) caused by SARS-CoV-2, is thought to spread mainly between people who are in close contact with one another within about 6 feet (1.8 m) when an infected person coughs, sneezes or talks.[1] Aerosol droplets containing the SARS-CoV-2 virus have been shown to remain suspended in air for ~3h.[2] These droplets can land in the mouths or noses of people who are nearby or possibly be inhaled into the lungs. It is important to discuss minimal infectious dose and viral load exposure. The minimal infective dose is defined as the lowest number of viral particles that cause an infection in 50% of individuals, The human infectious dose of SARS-CoV-2 remains unknown, but animal studies are a plausible surrogate.[3] Currently, there is no approved vaccine for the prevention of COVID-19 and medications are limited for treatment. In an attempt to decrease viral load exposure, the government initiated a stay at home order to help avoid transmission of COVID-19, but this could result in physical deconditioning if individuals become progressively more sedentary.

This is a new disease and there is limited information regarding risk factors for severe disease. Based on what we know now, the CDC classifies those at high-risk for severe illness from COVID-19 are people 65 years and older, BMI $\geq$ 40, diabetes, live in nursing home or long-term care facility, chronic lung disease, moderate asthma, heart conditions, or immoncompromised (cancer, organ transplant, HIV, smoking, prolonged corticosteroids, dialysis, liver disease.)[4] It is important to utilize exercise as medicine during this time, not only as an effective way to treat obesity, diabetes, or heart and lung disease that are considered to place a patient at high risk, but also utilizing exercise to precondition individuals prior to developing COVID-19. It is important to consider the significant decrease in cardiorespiratory fitness (CRF) and profound loss of muscle mass when patients are hospitalized for weeks at a time, especially if mechanical ventilation is required. After fighting for their life against COVID-19, at the time of discharge patients will have an ongoing battle to regain the CRF and strength that they lost during the illness. Hence, it is now even more important than ever to prophylactically increase CRF, muscular strength, and optimize body composition through exercise preconditioning.

The use of face masks have also been shown to be effective in decreasing transmission. A randomized controlled trial of 446 nurses comparing the use of surgical masks with the N95 respirator in

protecting health care workers against influenza found that use of a surgical mask compared with an N95 respirator resulted in noninferior rates of laboratory confirmed influenza.[5] In an observational study, researchers assessing the effect of community-wide mask usage to control COVID-19 observed 11 COVID-19 clusters in recreational "mask-off" settings compared to only 3 in workplace "mask-on" settings. Concluding that mask wearing may contribute to the control of COVID-19 by reducing the amount of emission of infected saliva and respiratory droplets from individuals with subclinical or mild COVID-19.[6]

The presence of gaps between the mask and the facial contours will result in air leakage around the mask (face seal leakage) reducing the effectiveness of the mask. .Researchers assessing the efficiencies of protection offered to by N95 respirator and surgical masks in 25 subjects to evaluate the 2 pathways of aersol particle penetration (filter vs fasce seal) into respiratory protection devices (RPDs). They concluded health care environments should be increasingly focused on the peripheral design rather than on the further improvement of the filter media. The faces eal leakage was found to represent the main pathway for the submicrometer particles penetrating into the mask. Thus, concluding that the priority in product development should be given to establishing a better fit that would minimize the faces eal leakage to increase protection for the individual wearing the mask.[7] In addition to being effective at decreasing the spread of the virus, a mask must be comfortable enough to be tolerated by the wearer to maximize compliance.

Konda et.al tested the performance of over 15 natural and synthetic fabrics to determine the best materials to use when making a cloth mask. They found that cotton sheets with high thread counts provide the best mechanical protection. However, other materials such as natural silk, chiffon weave or flannel can likely provide good electrostatic filtering of particles. Combining layers to form hybrid masks, leveraging mechanical and electrostatic filtering may be an effective approach. This could include high thread count cotton combined with two layers of natural silk or chiffon, for instance. A quilt consisting of two layers of cotton sandwiching a cotton−polyester batting also worked well. In all of these cases, the filtration efficiency was >80% for <300 nm and >90% for >300 nm sized particles. More importantly they also found that face seal leakage around the mask area can degrade efficiencies > 60%.[8]

Following the severe acute respiratory syndrome (SARS) outbreak that occured in March 2003, a study by Li et.al. was conducted to investigate the effects of wearing N95 and surgical facemasks by assessing the subjective perception of discomfort and heart rate response to each mask following a walking protocol. Subject completed 3 walking intervals as follows: 20 min at 2.0 mph (3.2 km/h), 10 min at 3.0 mph (4.8 km/h),10 min at 4.0 mph (6.4 km/h) and resting 10 min between each interval. Subjects rated ten sensations of discomfort: humidity, heat, breathing resistance, itchiness, tightness, saltiness, feeling unfit, odor, fatigue, and overall discomfort, and asked to reply to the question ‘‘How do you like the facemask?’’ by rating on a scale ranging from 0 to 10, with 0 representing ‘‘not at all’’, 5 representing ‘‘acceptable’’ and 10 representing ‘‘very fond of’’. In general, the ratings for humidity, heat, breath resistance and overall discomfort increased gradually with time and increase of workload. Surgical facemasks had significantly lower ratings than the N95 facemask, which suggested that when wearing either of the surgical facemasks the subject felt drier, cooler, more able to breathe easily and less uncomfortable than when wearing either of the N95 facemasks. The pattern of changes in mean heart rate amongst these facemasks is similar, reaching peaks at the end of the third exercise session. The subjects had lower mean heart rates when wearing surgical masks than when wearing N95 facemasks. High breathing resistance made it difficult for the subject to breathe and take in sufficient oxygen.[9]

Significance

The CDC currently recommends that everyone wear a cloth face covering (not surgical masks or N-95 respirators) in public, especially in places where maintaining social distancing is difficult to maintain (e.g., grocery stores and pharmacies).[10] These recommendations become increasing important as the country gradually begins to lift restrictions by opening more businesses including gyms and fitness centers. Johns Hopkins released a report, “Public Health Principles for Phased Reopening During COVID-19: Guidance for Governors” outlining how to re-open certain categories of activities including gyms and fitness centers while reducing COVID-19 risk. The report states that regardless of business specific considerations, there are measures that can be taken to mitigate the risk of infection to protect individuals. COVID-19 mitigation measures included the use of nonmedical cloth masks, incorporating engineering controls such as physical barriers where possible, and reconfiguring space to enable people to be located at least 6 feet apart.[11] The report

also ranks certain activities by their contact intensity, number of contacts, and the potential to modify them to reduce risk. Identifying gyms and fitness studios as “nonessential” businesses, rating contact intensity, number of contacts, and modification potential all as medium.[11] The purpose of a cloth mask is not to protect the individual wearing the mask, as much as it is to protect other people surrounding them by reducing the viral load shedding into the environment. If individuals are able to exercise at home or outside while practicing appropriate social distancing, then it is reasonable to not wear a mask. However, the COVID-19 pandemic can make it challenging to maintain a physically active lifestyle, and has created concern about exercising as gyms begin to open back up for business. Wearing a mask during exercise is going to impede the ease of breathing. Although decreased faceseal leakage results in improved protection, it increases airflow resistance and impede the ease of breathing, which becomes increasing important during exercise. This highlights the importance of assessing the risk vs benefits of the mask and finding the best mask for that specific exerciser. For high risk individuals it might be more beneficial to protect their self by wearing mask with minimal faceseal leakage. However, a mask with slightly more faceseal leakage might be more appropriate for individuals that are asymptomatic and exercising at a higher intensity. The CDC recommendation to wear a cloth face covering is to protect people around the individual wearing mask more than individual, which highlights the importance of everyone wearing a mask to protect the community.[12] Especially during times of increased viral shedding including talking, coughing, sneezing, or increased ventilation during physical activity.

This study intends to find out how a cloth mask may impact exercise capacity, to provide guidance for exercisers to adjust their expectations and training accordingly. We plan to asses exercise capacity through estimated peak oxygen consumption (eVO_2peak), oxygen saturation and level of perceived exertion during treadmill based exercise while wearing a cloth mask compared to exercising without a cloth mask. The potential significance of this study is to determine if subjects can exercise safely and if their exercise training needs to be adjusted while following the current recommendations of wearing a cloth mask in public. The degree of airflow limitation experienced will depend on the type and fit of the mask being worn, and inadequate airflow could possibly result in CO2 re-breathing if all air was not fully discharged from the mask with each breath. This re-breathing of CO2 could potentially limit the workload leading to a detriment in performance, and increase in

adverse symptoms such as dizziness, lightheadedness, chest pain or shortness of breath that does not improve with rest. Getting used to the mask may be difficult. Practice first with walking. Patients should be reminded not to panic if they are struggling with breathing with the mask on they should just lift up the mask for some extra air taking deep diaphragmatic breaths, and avoiding shallow breathing which can lead to hyperventilation. If we are going to recommend that individuals exercise with cloth masks, it is imperative that future research is conducted to evaluate the degree to which airflow can be limited during exercise by wearing a cloth mask. At the completion of our study, we will have further understanding of the effects of face masks during treadmill based running. To our knowledge this is the first study to assess the effects of wearing protective cloth mask on exercise capacity.

Hypothesis

Our null hypothesis H_O states that exercising with a cloth mask will be inferior to the current standard of exercising without a facemask, limiting peak exercise when non-inferiority margin is 1 estimated metabolic equivalent (eMET 3.5 $mlO_2*kg^{-1}*min^{-1}$).

Our alternative hypothesis H_1 states that exercising with a mask will not be inferior to the current standard of exercising without a facemask, limiting peak exercise when non-inferiority margin is 1 eMET.

Specific Aims

1) To gain further understanding on effects exercising with a cloth mask has on exercise capacity.

Our working hypothesis is that wearing a face mask will limit exercise capacity by a minimal clinically important difference (MCID) of 1 estimate metabolic equivalent (eMET).

EXPERIMENTAL DESIGN & METHODS

Subject Recruitment: We will recruit 20 subjects though the Cleveland Clinic. Inclusion Criteria: Healthy subjects age $\geq$ 18, Exclusion Criteria: Any long-term disease that would interfere with their ability to exercise safely, fever $\geq$100.4 F, or pregnancy. Informed consent will be obtained by the CoPI, and need for medical clearance determined based on ACSM's 2015 preparticipation guidelines.[13] (See appendix 12.6) To remain compliant with enterprise guidelines we will not be recruiting healthy subjects outside of the Cleveland Clinic staff at this time to avoid increase COVID-19 exposure risk. However, if the enterprise changes the guidelines we will expand our enrollment to include non Cleveland Clinic employees. Employees will not be solicited by

direct initiation. Recruitment will involve only posted notice or general advertising that does not pressure employees into participating for fear of job loss, delayed promotion, or other influences from their superior.

Study Design: The study design is a prospective crossover non-inferiority trial where subjects will complete 3 separate graded exercise treadmill tests each time wearing either No Mask, N-95 Mask (3M), or Cloth Mask (Boco). The order of the mask worn will be randomly assign to minimize familiarization or training effect. All testing will be performed in a Cleveland Clinic facility following standardized COVID-19 screening precautions. We will compare the data collected from each test to asses for differences between subject comfort, peak exercise capacity (eMET), heart rate response during exercise and recovery.

Testing: After completing the informed consent and being medically cleared[13], subjects will then perform a symptom limited graded exercise treadmill test using a modified Balke protocol. The modified Balke, which is a well-accepted treadmill protocol that keeps the speed constant and increases workload by grade. Subjects will rest for 3 minutes and resting heart rate, blood pressure, subjective perceptions of the face mask will be measured. Subjects then will walk at 0% grade at 3 miles per hour for 2 minutes. Elevation will be increased by 2% after the initial 2 minutes and by 1% each minute thereafter until the test is terminated. Heart rate will be monitor by Polar H10 heart rate monitor and oxygen saturation will be measured with a pulse oximeter.[14] We will continue to monitor heart rate during recovery at 1, 3 and 5 minutes post exercise. We will monitor each stage for perceived exertion, feelings of light headedness, anxiety, and discomfort. The test will also be terminated at the request of the subject or if any chest pain develops. The scale of measuring subjective perceptions instrument will be adminstereted after collecting 5 minutes of resting, end of the exercise test, and at 5 minutes post recovery. The graded exercise test will be performed once with No Mask, once with 3M 8200 N-95 Respirator, and once with a Cloth Mask (Boco). Subjects will be randomized by a random number generater (1= No Mask, 2= N-95, 3= Cloth Mask)[15] to determine the order the perform each exercise test No Mask, N-95 or Cloth Mask and will complete the other tests 2 atleast 1 day apart. Subjects will be instructed to abstain from exercise for 2 days before the test.[16]

Mask:

3M 8200 N-95 Respirator obtain from a third party provider. N-95 Respirators have been purchased outside the Cleveland Clinic supply chain. Cloth Face Mask manufactured by Boco is a custom designed two-layer face masks are built with a tightly woven polyester outer shell fabric and a soft, breathable performance knit mesh inner linig. Includes a slit pocket where a filter can be added.

COVID-19 Precautions

Subject

Temperature screening upon entering building to determine specific temp before denying subject access (100.4). COVID screening questions upon entry in to hospital. Questions to include whether subject has come from a household with a COVID + family/friend. Pending COVID test – deny entry until negative result determined. Sanitizers available per current placement on walls all patient areas. (Deemed adequate by HH IP department already). Subjects instructed on procedure for hand sanitizing. Subjects to wear cloth mask/hospital-provided mask upon entry and exit. Advised use during exercise as tolerated. Instruction on mask donning and doffing done prior to class start. Absolutely no visitors. Subjectss to be dropped off so as to limit number of people in department. Subjects 6 feet apart. Testing restricted to 39 minutes to accommodate equipment sanitation, education, flow of people entering and leaving. Should subjects use towels, they must be provided by hospital, used solely by subject and disposed of in appropriate hamper by subject. Directional flow signage to/from waiting area-gym-conference room. Subjects to be provided individual baggies with supply of masks. Monitors are assigned to each subject, sanitized before and after subject use.

Investigators:

Investigators are to wear cloth or surgical mask at all times. Staff to wipe down equipment between each subject use using recommended disinfectant wipes. No food or drink in patient care areas. Staff will carry individual sanitizers for individual or subject use.

Infection Prevention:

Equipment wiped off between each subject's use with Super Sani-Cloth germicidal disposable wipes. Polar heart rate monitors and straps will be immediately wiped down with Super Sani-Cloths, and then all straps

will be washed per manufacturers recommendations. Reusable cuffs wiped between classes. Stethoscope disinfected with alcohol between patient. Pulse oximeters wiped with alcohol between each subject use. No equipment with fans to be used; includes Air Dyne bike and rower. No locker use, Restrooms limited to no more than 2 individuals at a time and signage added to restroom doors to notify of 2 person limit, with and washing instruction applied to mirrors. Exercise equipment spaced 6 feet apart.

Statistical Analysis

<u>Non-inferiority design:</u> $H0$: μMask — μNoMask $\leq$ — δ vs. $H1$: μMask — μNoMask > — where $\delta > 0$ denote the non-inferiority margin of 3.5, which is a (clinically meaningful) minimal detectable difference. <u>The sample sizes are</u>: Total sample size n= 20 nNoMask = (1 + 1/k) * σ2 * ((Z1 — α + Z1 — β)/ (d + δ))2nMask = k * nNoMask where σ2 is the variance, and d = μMask — μNoMask is known as the allowable difference, which is the true mean difference between the new treatment (Mask) and the control (NoMask). 1) δ = 3.5$mlO_2 * kg^{-1} * min^{-1}$(1 eMET=3.5 $mlO_2 * kg^{-1} * min^{-1}$): a (clinically meaningful) minimal detectable difference 2) k=1 : the ratio of the sample size of treatment(mask) group to the sample size of control(NoMask) group 3) σ2= 3.5: population eMET change variance 4) power: 0.9 5) 5% expected rate of drop-outs 6) d=0: Descriptive statistics will be used to characterize the patient cohort. Demographic variables will be described using mean with standard deviations for continuous variables and counts with percentages for categorical variables. The Wilcoxon signed-rank signed test or paired-sample t-test will be used to compare continuous variables between the Mask and NoMask exercise. McNemar's test will be applied to compare categorical variables. Mixed-effected linear regression models will be applied for the repeated measures data. Pearson correlation coefficient will be calculated whenever it is appropriate. Analyses will be two-tailed and performed at significance level of 0.05 with SAS 9.3 software (SAS Institute, Cary, NC).

Project Timeline

All testing expected to be completed within 2 weeks, followed by data and statistically analysis. We intend to have this study written up and published prior to the opening of public gyms in Ohio.

Budget

Funding by Joseph Parambil and Pulmonary Institute

Personnel

Principal Investigator (CCF): Debasis Sahoo MD will serve as an investigator assisting with the study design and analysis of data.
Co-Investigator (CCF): Matthew Kampert MS, DO will serve as an investigator and Exercise Physiologist conducting exercise testing for this study. He will coordinate the overall scope of the project, recruit participants, and oversee data collection as well as analysis, presentation, and publication.

Co-Investigator (CCF): Tamanna Singh, MD

Co-Investigator (CCF): Richard Figler, MD

Biostatisticians (CCF): Han Xiaozhen and Xiaofeng Wang assist with statistical design and analysis of data.

References

1. How Coronavirus Spreads | CDC. https://www.cdc.gov/coronavirus/2019-ncov/prevent-getting-sick/how-covid-spreads.html.
2. van Doremalen, N. *et al.* Aerosol and Surface Stability of SARS-CoV-2 as Compared with SARS-CoV-1. *The New England journal of medicine* vol. 382 1564–1567 (2020).
3. ST-Master Question List for COVID-19 | Homeland Security. https://www.dhs.gov/publication/st-master-question-list-covid-19.
4. People Who Are at Higher Risk for Severe Illness | CDC. https://www.cdc.gov/coronavirus/2019-ncov/need-extra-precautions/people-at-higher-risk.html.
5. Mahony, J. *et al.* Surgical Mask vs N95 Respirator Among Health Care Workers. **302**, 1865–1871 (2009).
6. Cheng, V. C.-C. *et al.* The role of community-wide wearing of face mask for control of coronavirus disease 2019 (COVID-19) epidemic due to SARS-CoV-2. *J. Infect.* (2020) doi:10.1016/j.jinf.2020.04.024.
7. Grinshpun, S. A. *et al.* Performance of an N95 filtering facepiece particulate respirator and a surgical mask during human breathing: Two pathways for particle penetration. *J. Occup. Environ. Hyg.* **6**, 593–603 (2009).
8. Konda, A., Prakash, A., Moss, G. A., Schmoldt, M. & Grant, G. D. Fabrics Used in Respiratory Cloth Masks. (2020) doi:10.1021/acsnano.0c03252.
9. Li, Y. *et al.* Effects of wearing N95 and surgical facemasks on heart rate, thermal stress and subjective sensations. *Int. Arch. Occup. Environ. Health* **78**, 501–509 (2005).
10. Recommendation Regarding the Use of Cloth Face Coverings | CDC. https://www.cdc.gov/coronavirus/2019-ncov/prevent-getting-sick/cloth-face-cover.html.
11. Rivers, C. *et al.* Public Health Principles for a Phased Reopening During COVID-19: Guidance for Governors 2 AUTHORS. (2020).
12. Cloth Face Coverings: Questions and Answers | CDC. https://www.cdc.gov/coronavirus/2019-ncov/prevent-getting-sick/cloth-face-cover-faq.html.
13. Riebe, D. *et al.* Updating ACSM's recommendations for exercise preparticipation health screening. *Med. Sci. Sports Exerc.* **47**, 2473–2479 (2015).
14. Gillinov, S. *et al.* Variable Accuracy of Wearable Heart Rate Monitors during Aerobic Exercise. 1697–1703 (2017) doi:10.1249/MSS.0000000000001284.
15. random number generator - Google Search. https://www.google.com/search?q=random number generator&hy_es_tkn=nv0wfzhl.
16. Katch, Biological variability in maximum aerobic power. MSSE 1982 (1).pdf.

Appendix

ACSM's 2015 preparticipation health screening guidelines[13]

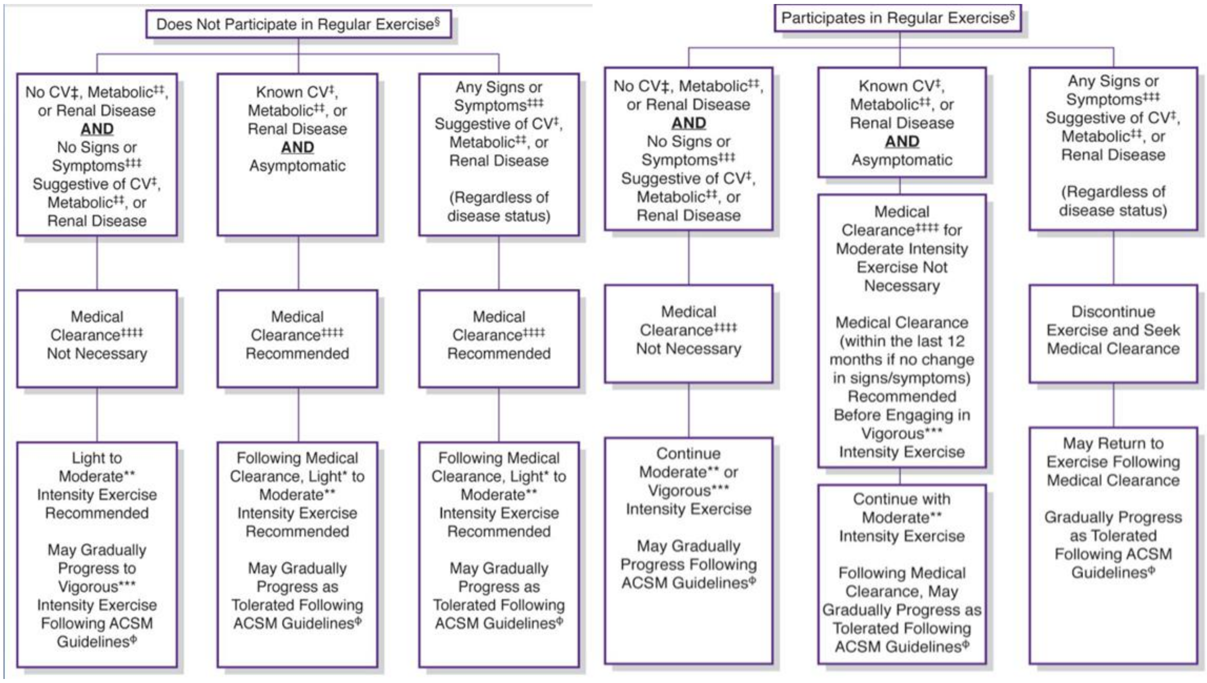

Step 1

SYMPTOMS
Does your client experience:

____ chest discomfort with exertion
____ unreasonable breathlessness
____ dizziness, fainting, blackouts
____ ankle swelling
____ unpleasant awareness of a forceful, rapid or irregular heart rate
____ burning or cramping sensations in your lower legs when walking short distance

If you **did** mark any of these statements under the symptoms, **STOP**, your client should should seek medical clearance before engaging in or resuming exercise. Your client may need to use a facility with a **medically qualified staff**.

If you **did not** mark any symptoms, continue to steps 2 and 3.

Step 2

CURRENT ACTIVITY
Has your client performed planned, structured physical activity for at least 30 min at moderate intensity on at least 3 days per week for at least the last 3 months?

Yes ☐ No ☐

§Exercise Participation	Performing planned, structured physical activity at least 30 min at moderate intensity on at least 3 d · wk⁻¹ for at least the last 3 mo
*Light Intensity Exercise	30%–39% HRR or $\dot{V}O_2R$, 2–2.9 METs, RPE 9–11, an intensity that causes slight increases in HR and breathing
**Moderate Intensity Exercise	40%–59% HRR or $\dot{V}O_2R$, 3–5.9 METs, RPE 12–13, an intensity that causes noticeable increases in HR and breathing
***Vigorous Intensity Exercise	≥60% HRR or $\dot{V}O_2R$, ≥6 METs, RPE ≥14, an intensity that causes substantial increases in HR and breathing
‡Cardiovascular (CV) Disease	Cardiac, peripheral vascular, or cerebrovascular disease
‡‡Metabolic Disease	Type 1 and 2 diabetes mellitus
‡‡‡Signs and Symptoms	At rest or during activity. Includes pain, discomfort in the chest, neck, jaw, arms, or other areas that may result from ischemia; shortness of breath at rest or with mild exertion; dizziness or syncope; orthopnea or paroxysmal nocturnal dyspnea; ankle edema; palpitations or tachycardia; intermittent claudication; known heart murmur; unusual fatigue or shortness of breath with usual activities.
‡‡‡‡Medical Clearance	Approval from a health care professional to engage in exercise
ΦACSM Guidelines	See *ACSM's Guidelines for Exercise Testing and Prescription, 10th edition, 2018*

Scale of measuring subjective perceptions Instruments[9]

	Not at all	Mildly	Strongly
Humid			
Hot			
Breathe resistance			
Itchy			
Tight			
Salty			
Unfit			
Odour			
Fatigue	0 1 2 3	4 5 6	7 8 9 10

	Comfortable	Uncomfortable	Extremely Uncomfortable
Overall Discomfort	0 1 2 3	4 5 6	7 8 9 10
