# Supplementary material for: Effects of Wearing an N95 Respirator or Cloth Mask Among Adults at Peak Exercise: A Randomized Crossover Trial
Source: JAMA Netw Open. 2021 Jun 30;4(6):e2115219. doi: 10.1001/jamanetworkopen.2021.15219 (PMC8246308; doi:10.1001/jamanetworkopen.2021.15219)
Supplement: Supplement 2. — eAppendix. Supplemental Methods [file jamanetwopen-e2115219-s002.pdf]

Supplemental Online Content

Kampert M, Singh T, Sahoo D, Han X, Van Iterson EH. Effects of wearing an N95 respirator or cloth mask among adults at peak exercise: a randomized crossover trial. *JAMA Netw Open.* 2021;4(6):e2115219. doi:10.1001/jamanetworkopen.2021.15219

eAppendix. Supplemental Methods

This supplemental material has been provided by the authors to give readers additional information about their work.

© 2021 Kampert M et al. *JAMA Network Open.*

eAppendix. Supplemental Methods

Participants

We prospectively recruited and enrolled 20 never smoker asymptomatic and apparently healthy recreationally active men and women currently employed by the Cleveland Clinic to voluntarily participate in this study. Voluntary participation of individuals external to the Cleveland Clinic was prohibited due to institutional policies mandating COVID-19 related human-to-human research not involve unnecessary onsite medical testing and/or research procedures at any Cleveland Clinic facility.

Study inclusion criteria required potential participants be apparently healthy without history of any chronic disease (including absence of exercise induced asthma), asymptomatic and without previous positive COVID-19 test, demonstrate the ability to exercise on a treadmill without assistance, no orthopedic limitation that could be the cause of exercise termination, and ability to independently provide verbal and written informed consent. Patients were excluded if at the time of recruitment they were symptomatic, had a fever ≥100.4 F, or were pregnant. Each study participant provided voluntary verbal and written informed consent prior to participating in any aspect of this study. The Cleveland Clinic Institutional Review Board reviewed and approved all aspects of this study, including the assurance that this study was in full compliance with the Declaration of Helsinki.

Study Design

This was a prospective study conducted using a randomized-control crossover design where participants completed three treadmill EST experimental conditions each

© 2021 Kampert M et al. *JAMA Network Open.*

performed to volitional fatigue and on separate days with at least 48 hours and no greater than seven days between tests. Participants were instructed to abstain from caffeine ingestion (>12 hours) and performing exercise <24 H prior to testing, with the exception of exertion associated with activities of daily living.[9] The three experimental conditions performed by each participant, included EST with nm, n95 (M 8200 N-95 Respirator), and cm (Boco with PM2.5 activated carbon filter). To minimize the possibility of familiarization or training effects, a random number generater was used per participant to determine the order in which EST conditions would be performed.

To avoid utilizing personal protective equipment dedicated for patient care purposes, n95 masks were purchased outside the Cleveland Clinic supply chain from a third party vender, and were not fit tested. The cm manufactured by Boco is a two-layer face mask built with a tightly woven polyester outer shell fabric and a soft breathable performance knit mesh inner lining containing a slit pocket where a PM2.5 activated carbon filter was utilized in this study.

The EST protocol performed by each participant was an individualized modified-ramp Balke, whereby participants maintained a constant self-selected speed throughout the test while the grade increased beginning at the second minute from 0.0 to 2.0 %; and thereafter increased in a fixed increment of 1.0 % every minute until volitional fatigue. The self-selected treadmill speed was kept constant for each crossover condition. Throughout each EST, participants were monitored continuously using pulse oximetry to estimate arterial oxyhemoglobin saturation (SpO_2); and a 12-lead electrocardiogram was used to acquire beat-to-beat heart rate (HR) and rhythm. Measurements of HR, rating of perceived exertion (RPE; Borg scale, 0-10), and SpO_2 were acquired at baseline, during

© 2021 Kampert M et al. *JAMA Network Open.*

the last 5-10 seconds of each stage, and at 1, 3 and 5 minutes post exercise recovery. Study personnel verbally encouraged participants to provide a maximal effort and exercise until they could not go on any longer. Peak exercise exhaustion was verified if two or more of the following criteria were met: 1) HR within 10 bpm of age-predicted maximum HR (220-age), 2) RPE > 9, and/or 3) a plateau in HR (<3 bpm change over the last two intensity stages).

In addition to RPE scoring, the subjective experience of wearing or not wearing a facial covering was evaluated at both rest and within the immediate post-exercise recovery period using a perceptions instrument questionaire (see appendix for full list of items and scoring associated with this questionnaire).[4,10]

Relative and absolute clinical criteria that were referenced in order to determine whether any EST required immediate termination due to safety related concerns came directly from joint recommendations written by the American Heart Association, American College of Cardiology, and American College of Sport Medicine.[8] These criteria are institutionally considered clinical standard of care best practices and can be viewed in full detail elsewhere.[8]

Derived EST variables

Due to institutional COVID-19 policies, use of the clinical cardiopulmonary exercise testing lab and indirect calorimetry system was (and remains) reserved strictly for patient care and clinically indicated reasons in order to keep both patient and staff exposure risk low and to preserve resources and supplies. Therefore, to quantify aerobic exercise capacity we used a validated treadmill equation for estimating peak exercise oxygen uptake ($\dot{V}O_{2peak}$) as follows: speed × (0.17 + grade × 0.79) + 3.5; where speed is that of

© 2021 Kampert M et al. *JAMA Network Open.*

the belt in meters per min and grade is that of the belt platform as a fraction representing the terminal stage.[11] In instances where only a partial stage was completed before maximal voluntary exhaustion was achieved, we only considered those as peak exercise stages in $\dot{V}O_{2peak}$ calculations if at least 25 % of the stage was completed.

The percentage achieved of predicted $\dot{V}O_{2peak}$ (%$\dot{V}O_{2peak}$) was also calculated using sex, age, and body weight specific equations based on the dataset used for deriving the above $\dot{V}O_{2peak}$ equation.[11,12] Additionally, since predicted metabolic equivalents (METS) of task performed for peak exercise is derived differently than that of predicted $\dot{V}O_{2peak}$, but also used clinically, we calculated percentage achieved of predicted METS (%METS) based on sex and age.[13]

Because the never smoker healthy participants who participated in this study were recreationally active[14] and without diseases of either the cardiovascular or pulmonary systems, and acute or chronic conditions of the blood were also absent (e.g., anemia), average sex specific hemoglobin content and concentration levels reported for apparently healthy and similarly aged adults of the general US population were used herein for the calculation of estimated arterio-venous oxygen content difference ($Ca\text{-}vO_2$) at peak exercise.[15-17] Calculated $Ca\text{-}vO_2$ values also accounted for arterial oxygen saturation levels approximated via acquired SpO_2, an assumed (terminal) peak exercise mixed-venous oxygen saturation level of 24 %, and the expected change in hemoconcentration that occurs from rest to peak exercise.[16-18] Moreover, because of known relationships intrinsic to the oxygen dissociation curve, the effects from any decrement (per 1 %) in SpO_2 that occurred from a standard normal level of 96 % were also accounted for in Ca-

© 2021 Kampert M et al. *JAMA Network Open.*

vO_2 calculations.[16-19] Finally, the Fick principle was used to calculate peak exercise cardiac output ($\dot{Q}$) and all three components were plotted for each condition in Figure 1.[18] The exercise stage-to-stage HR response for each condition was fit to a zeroth-order growth kinetics model, $[HR_{peak}] = [HR_0] + [HRk]\times[stage]$; where we solved for the HR reaction rate constant k (HRk) as means to evaluate and compare the chronotropic phenotype between conditions. This approach was used to evaluate chronotropy as a dynamic-time dependent response (i.e., cardioacceleration) as opposed to a traditional cross-sectional response because it is necessary for this study to account for the possible autoregressive effects each metabolic transition from low- to- high exercise intensities can have on an eventual terminal HR. Because it is also known that HR demonstrates a steady-state linear rise as a function of fixed external work-rate associated with ramp testing, any deviation of contiguous HR responses from this expected chronotropic pattern would result in a HRk that could not be appropriately fit using a zeroth-order function. Therefore, having to employ the next higher order function to fit exercise stage-to-stage HR data to solve for HRk for a given experimental condition would signal the presence of a unique chronotropic and cardioacceleration phenotype.

The HRk parameter was also used in the plot of $\dot{V}O_{2peak}$ as a function of HR_{peak} in Figure 2 to confirm isopleths of oxygen pulse (O_2pulse) could be used to evaluate to what extent the change in derived $\dot{V}O_{2peak}$ corrected for SpO_2 tracked the observed linear change in stage-to-stage HR in order to estimate stroke volume (SV).[20-22] Despite not acquiring measurements of $\dot{V}O_2$ or SV, this approach for visualizing SV using O_2pulse isopleths was possible since the limit definition, $\lim_{n\to x}\left(\frac{f(x)-f(n)}{x-n}\right) = f'(x)$, is used to solve for the slope of the tangent line for a curve described by the function, $y = f(x)$, and because the

rise in O_2pulse ($\dot{V}O_2 \div HR$) as a function of linearly increasing HR can be described as a hyperbolic curve given the linear rise in $\dot{V}O_2$ as a function of increasing HR demonstrates a positive x-intercept as illustrated in Figure 2. Moreover, although we recognize it is assumed when deriving O_2pulse that CaO_2 remains relatively constant and that we had no way of confirming this factor in this study, it is also relevant that as HR increases linearly the effect of mixed-venous O_2 content on the variability in CaO_2 and oxygen transport becomes constant (and negligible) at peak exertion. As such, differences in aforementioned derived Ca-vO_2 that accounted for relevant drops in SpO_2 could be used to approximate the drop in CaO_2 that occurred in either of the two mask conditions as compared to the control nm condition. A resulting $\dot{V}O_{2peak}$ corrected for relevant decreases in SpO_2 for masked conditions relative to nm could then be calculated and plotted over O_2pulse isopleths in Figure 2.

The chronotropic response to submaximal levels of stress were evaluated as observed HR responses corresponding to stage #2 of exercise and roughly 65-75 % of heart rate reserve (HRR) across participants and conditions.[23,24] This allowed us to assess for mask worn exercise transients whether there was an inappropriate rise in HR at submaximal intensities approximately corresponding to metabolic domains associated with low-to-medium reliance on substrate-level phosphorylation for energy production.[24-26]

Statistical Analysis

Continuous data are reported as means±SD. Categorical/ordinal data are reported as n and percentage of sample or median (q25, q75). Mixed effects model ANOVA tests were performed to evaluate the overall effect of mask condition on continuous variables of interest where a normal distribution could be assumed. The restricted maximum

likelihood method was used to estimate the variance components for such models. By contrast, for dependent variables requiring no assumptions of the underlying data structure (e.g., perception scores), nonparametric mixed effects model ANOVA tests were performed where response data were fit using the minimum norm quadratic unbiased estimator method after being transformed to fit a rank-order structure.[27] The randomized order of mask condition was set as the random effect in all mixed effect ANOVA models. Post-hoc testing while correcting for multiple comparisons was performed to evaluate pairwise differences between conditions only for variables where the overall effect of mask condition was significant.

Additional mixed effects model ANCOVA tests were performed to evaluate the overall effect of mask condition on $\dot{V}O_{2peak}$ adjusted for 'breathing resistance' associated with mask wearing at peak exercise listed on the perceptions instrument questionaire. To avoid the possible effects of multi-collinearity in ANCOVA tests, other variables were excluded and only 'breathing resistance' was considered as a covariate since this is the only perception variable that would logically have the potential to yield an early exercise terminating effect and not result in high frequency false-positive elevated scores provided for the nm EST transient.

Two-tailed significance was determined using an alpha level set at 0.05. Analyses were performed using SAS statistical software v.9.4 (SAS Institute, Cary, NC).

© 2021 Kampert M et al. *JAMA Network Open.*

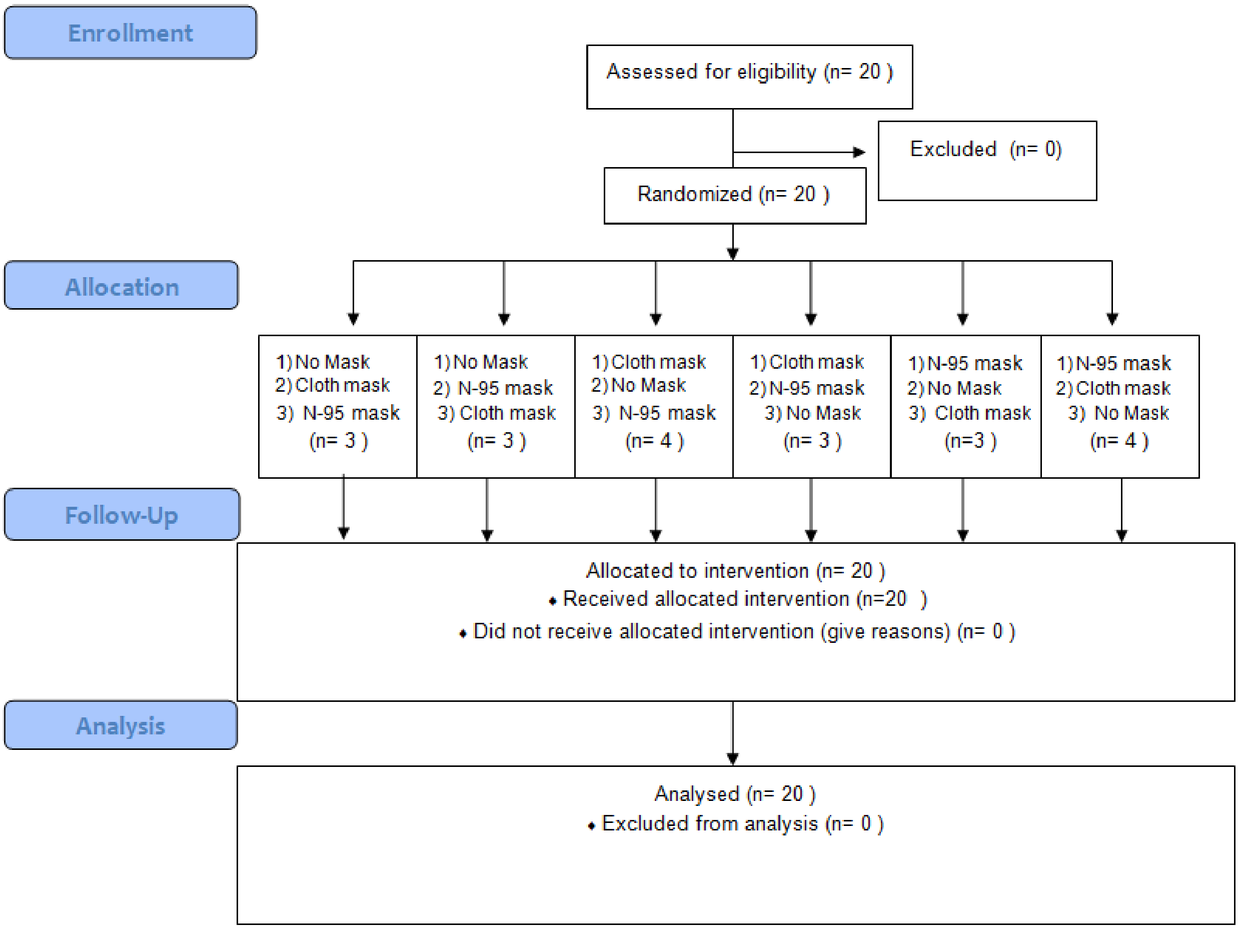

© 2021 Kampert M et al. *JAMA Network Open.*
