# Supplementary material for: Effects of Wearing an N95 Respirator or Cloth Mask Among Adults at Peak Exercise: A Randomized Crossover Trial
Source: JAMA Netw Open. 2021 Jun 30;4(6):e2115219. doi: 10.1001/jamanetworkopen.2021.15219 (PMC8246308; doi:10.1001/jamanetworkopen.2021.15219)
Supplement: Supplement 3. — Data Sharing Statement [file jamanetwopen-e2115219-s003.pdf]

Data Sharing Statement

Kampert M, Singh T, Sahoo D, Han X, Van Iterson EH. Effects of wearing an N95 respirator or cloth mask among adults at peak exercise. *JAMA Netw Open.* 2021;4(6):e2115219. doi:10.1001/jamanetworkopen.2021.15219

Data
Data available: No
